# Wild edible ectomycorrhizal fungi: an underutilized food resource from the rainforests of Tshopo province (Democratic Republic of the Congo)

**DOI:** 10.1186/s13002-020-0357-5

**Published:** 2020-02-10

**Authors:** Héritier Milenge Kamalebo, André De Kesel

**Affiliations:** 1 Faculté des Sciences, Université de Kisangani, BP 2012, Kisangani, Democratic Republic of the Congo; 2 Centre de Recherches Universitaires du Kivu (CERUKI-ISP), BP 854, Bukavu, Democratic Republic of the Congo; 3 Meise Botanic Garden, Nieuwelaan 38, Meise, Belgium

**Keywords:** Edible ectomycorrhizal fungi, Fungal consumption, Tshopo, Democratic Republic of the Congo

## Abstract

**Background:**

Ectomycorrhizal (EcM) fungi constitute a source of income as well as proper food with considerable nutritional value. Although edible EcM fungi are highly diverse and expected to host considerable nutritional attributes, only few studies focus on their use and promotion in the province of Tshopo (DR Congo). This study provides original ethnomycological and diversity data on edible ectomycorrhizal rainforest fungi from the Man-and-Biosphere reserve of Yangambi and the reserve of Yoko.

**Methods:**

The list of edible fungi follows the current taxonomy. Taxa were collected in plots situated in different types of rainforests. Each taxon is supported by herbarium reference specimens. Ethnomycological data on locally consumed EcM fungi were collected from randomly selected people living near the Man-and-Biosphere reserve of Yangambi and the Yoko reserve. People were interviewed using a semi-structured questionnaire. The interview campaign involved 160 informants, all randomly selected from 6 different ethnic communities.

**Results:**

The results reveal that rainforests from the Yangambi Biosphere reserve and Yoko forest reserve provide a relatively high number of edible fungi, more than local people actually use. Mixed forest stands hold the highest diversity in saprotrophic edible fungi (*p* value < 0.001) while no significant difference (*p* value > 0.05) was observed in the number of saprotrophic and EcM fungi within monodominant forests. In spite of being accessible, this renewable natural resource is underexploited. Although a wide array of EcM fungi is available in primary forests dominated by ectomycorrhizal trees, local people’s major interest goes to the saprotrophic fungi from areas with degraded mixed forests.

**Conclusion:**

The lack of local interest for EcM fungi is probably related to the considerable distance people have to cover to collect them. As a result, the edible EcM fungi from the Tshopo area represent a potentially interesting but underutilized resource.

## Introduction

In many regions of the world, including tropical Africa, edible fungi often constitute a source of income and food, with a non-negligible nutritional value [1–6]. According to De Kesel et al. [4] and Degreef et al. [7], documenting the relative importance and potential of locally used EcM fungi remains vital for improving strategies for their conservation and promoting sustainable use. Edible EcM fungi provide high-quality and important amount of crude protein, minerals and carbohydrates, fats, etc. [2, 3, 5, 6]. The amount of fungal crude protein for instance is generally ranked between 19 and 35% of the dry mass [2]. Furthermore, many species of edible EcM fungi provide higher amount of unsaturated fatty acids than saturated ones [2, 5]. Unlike saturated fatty acids (found in high amount in animal fats), unsaturated fatty acids are suggested to be essential in the human diet [2]. The high proportion of unsaturated fatty acids and high percentage of linoleic acid found in edible EcM mushrooms lead to consider them as a healthy food [2]. In addition, the proteins of most of edible EcM taxa contain all nine essential amino acids (leucine, isoleucine, valine, tryptophan, lysine, threonine, phenylalanine, methionine, and histidine) used in humans’ nutrition [2, 5].

EcM fungi constitute one of the most important groups of edible fungi worldwide. In tropical Africa, EcM fungi are massively consumed in the Zambezian region [4, 5, 8–14], and in the savanna woodlands and open forest areas of the Soudanian region [15–17]. Within the Zambezian geographical zone, local people collect, sell, and consume impressive quantities of EcM taxa. The socio-economic interest for EcM fungi is considered a major incentive for obtaining strong and broadly supported forest conservation programs [4].

Within the rainforests of Tshopo province (Democratic Republic of the Congo), many species of edible EcM fungi are available. In this area, EcM fungi are typically found within primary forests dominated by EcM partner trees such as *Gilbertiodendron dewevrei* (De Wild.) J. Léonard, *Brachystegia laurentii* (De Wild.) Louis, *Julbernardia seretii* (De Wild.) Troupin, *Uapac*a *guineensis* Müll. Arg, and *U. heudelotii* Baillon [18, 19]. This study aims to assess the distribution and diversity of edible fungi within various types of rainforests found in the Man-and-Biosphere reserve of Yangambi and the Yoko reserve. We show how access and availability, as well as trophic level of fungi, affect the interest of local people, and how this in turn creates or annihilates opportunities for forest conservation.

## Materials and methods

### Study site and sampling of fungi

Fieldwork was done in the province of Tshopo, located 2° N 2° S and 22° E 28° E [20]. The vegetation from Tshopo is mainly characterized by a tropical evergreen rainforest and some groves of semi-deciduous forests found on hills and plateaus [20–22]. These rainforests are mainly dominated by species such as *Gilbertiodendron dewevrei* (De Wild.) J. Léonard, *Brachystegia laurentii* (De Wild.) Louis, *Scorodophloeus zenkeri* Harms, *Prioria balsamifera* (Vermoesen) Breteler, and *Julbernardia seretii* (De Wild.) Troupin [20–24].

**Fig. 1:**
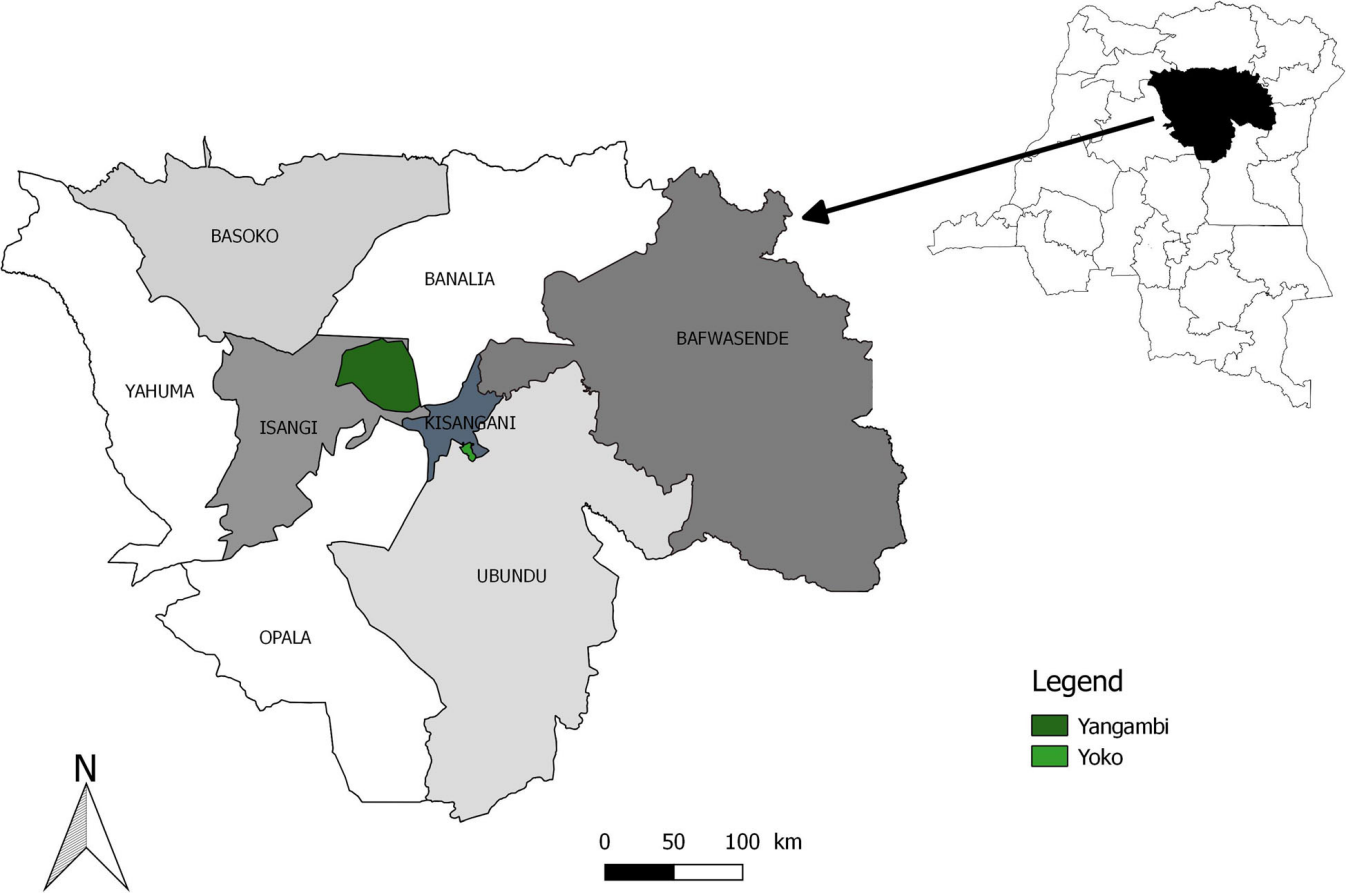
Location of the study area

The fungi were collected between 2013 and 2016, mostly within plots situated in the rainforests of the Biosphere reserve of Yangambi (0° 51′ 01.62″ N; 24° 31′ 43.53″ E) and Yoko reserve (0° 17′ 34.9″ N; 25° 18′ 27.4″ E) (Fig. 1). Monitoring of plots follows Lodge et al. [25], and fungal sampling was performed within forests dominated by *Gilbertiodendron dewevrei*, *Brachystegia laurentii*, *Julbernardia seretii*, *Uapaca heudelotii*, and *Uapaca guineensis* and in mixed forests. Three plots of 100 × 100 m were demarcated in each type of forest; each of them divided in a 20 × 20 m grid. Due to the elongated shape of *Uapaca heudelotii*-dominated forests, more stretched plots were installed in this forest type. In each plot, the aboveground fruiting bodies of all EcM fungi were collected by walking parallel bands covering the entire plot [26]. Unidentifiable fruit bodies were photographed in situ and dried after notes were taken from their macromorphological features (following [18]). Voucher specimens were dried using a field drier [27] and deposited at the herbarium of Meise Botanic Garden (Belgium).

### Fungal identification

The identification of voucher specimens was done using macroscopic and microscopic characteristics, as outlined in Eyi-Ndong et al. [18]. The available taxonomic literature only covers a fraction of the Central African rainforest fungi. The following contributions were used for identification: Heinemann [28], Heim [29], Pegler [30], Heinemann and Rammeloo [31–33], Buyck [8, 34, 35], De Kesel et al .[4, 9, 16], Verbeken and Walleyn [36], Eyi-Ndong et al. [18], as well as identification keys provided by the Fungus Flora of Tropical Africa (https://www.ffta-online.org/) and Edible Fungi of Tropical Africa (https://www.efta-online.org/). Species names and author’s abbreviations largely follow Index Fungorum (http://www.indexfungorum.org/Names/Names.asp). Unidentified taxa were left out of the analysis.

### Ethnomycological data acquisition and treatment

Ethnomycological data on locally consumed fungi were collected from local people living around the Man-and-Biosphere reserve of Yangambi and the Yoko reserve. Data were collected using open, semi-structured interviews (paper fill-in questionnaires). The interviews involved mainly the head of the family, sometimes assisted by other family members. The questions focused basically on the informant’s knowledge concerning the different locally consumed edible fungi. Interviews were obtained from 160 informants, all randomly selected, but belonging to one of 6 ethnic communities (Bakumu, Turumbu, Topoke, Lokele, Ngelema, and Ngando). The entire pool of informants counted 88 men (55%) and 72 women (45%), ranging from 16 to 72 years old. The interviewed communities live in four villages in the vicinity of the Man-and-Biosphere reserve of Yangambi (Yakako, Yalungu, Lyoli, and Lobiloto), 4 suburbs of Yangambi city (IFA, Lusambila, Ekuchu, and Manzikala), and 4 villages surrounding the Yoko forest reserve (Babogombe, Biaro, PK 48, and PK 25).

With an average of 600 households living in the studied area, the average number of studied households revolves around 25 per village (4.2% of the entire pool). Within the 4 suburbs of Yangambi, the distribution of households was 150 (25%), 120 (20%), 80 (13.3%), and 50 (8.3%) respectively reported from Ekuchu, Lusambila, Manzikala, and IFA. According to Gumucio et al. [37], the sample size (*n*) of the interviewed households should be calculated as follows: *n* = $\frac{N}{1+N\times {e}^2}$, where *N* is the total number of available households and *e* is the level of precision. With a precision level of ± 7%, the sample size was calculated by the following formula: *n* = $\frac{600}{1+\left(N\times {0.07}^2\right)}$ ≈ 152, households that were fitted to 160 informants. Referring to the mean distribution of households per sampling site (villages and suburbs of Yangambi), 7 households were interviewed from each of the 4 villages while 38 from Ekuchu, 30 from Lusambila, 20 from Manzikala, and 12 households from IFA. In each sampling site, all households were numbered. The first numbers referring to the considered sample size were selected randomly using the function “rand.between” of the Excel software.

The analyzed data only refer to locally eaten fungi and allow to present (per species) information on the edible mushrooms’ cultural significance (EMCS). According to Pieroni [38], the edible mushrooms’ cultural significance index refers to the importance or the role that a given fungal taxon or group of fungi plays in the social life of a group of people or a community. Using pictures or fresh sporocarps of edible fungi, the edible mushrooms’ cultural significance for a fungal species corresponds with the sum of the scores for “edibility status” given by all informants, divided by the total number of informants. Edibility status scores or frequencies of mention (FM) were assigned through informants’ answers to the following question: Do you eat this mushroom? (yes = 1, no = 0). A Kruskal-Wallis analysis was used to test the role of ethnicity on the edibility status score, and how this changes according to the different trophic groups.

## Results

### Checklist and diversity of wild edible fungi

Table 1 presents the checklist of edible fungi recorded from the rainforests of Tshopo. Based on the information found in the literature and ethnomycological inquiries, 78 species of macrofungi are edible.

**Table 1:** List of edible fungi from rainforests of Tshopo

Species	Edibility status	Trophic group	FM	EMCS score	Supporting literature	Voucher specimens
1. *Agaricus bambusicola* Heinem.	Food	Sapr	24	0.2	Milenge et al. [39]	ADK5267
2. *Agaricus crocopeplus* Berk. & Broome	Food	Sapr	27	0.2	Milenge et al. [39]	ADK5747, MKH073
3. *Amanita annulatovaginata* Beeli	Food	EcM	41	0.3	Milenge et al. [39]	MKH016
4. *Amanita echinulata* Beeli	Food	EcM	35	0.2	Milenge et al. [39]	ADK5938, MKH159
5. *Amanita pudica* (Beeli) Walleyn	Food	EcM	49	0.3	Degreef et al. [7], De Kesel et al. [4], Milenge et al. [39]	ADK5924
6. *Amanita robusta* Beeli	Food	EcM	49	0.3	Boa [1], Milenge et al. [39]	ADK5236
7. *Armillaria heimii* Pegler	Food	Sapr	78	0.5	Eyi-Ndong et al. [18], Degreef et al. [7], Milenge et al. [39]	ADK5230, MKH046
8. *Auricularia cornea* Ehrenb.	Food	Sapr	144	0.9	De Kesel et al. [16], Boa [1], Eyi-Ndong et al. [18], Degreef et al. [7], De Kesel et al. [4], Milenge et al. [39]	ADK5175
9. *Auricularia delicata* (Mont.ex Fr.) Henn.	Food	Sapr	142	0.9	De Kesel et al. [16], Boa [1], Degreef et al. [7], De Kesel et al. [4], Milenge et al. [39]	ADK5169, MKH214
10. *Calyptella longipes* (Cooke & Massee) W.B. Cooke	Food	Sapr	12	0.1	Milenge et al. [39]	ADK5868
11. *Cantharellus congolensis* Beeli	Food	EcM	89	0.6	De Kesel et al. [16], Boa [1], Eyi-Ndong et al. [18], Degreef et al. [7], De Kesel et al. [4], Milenge et al. [39]	ADK5199, MKH180
12. *Cantharellus conspicuus* Eyssartier, Buyck & Verbeken	Edible	EcM	0	0.0	Eyssartier et al. [40]	ADK5937
13. *Cantharellus densifolius* Heinem.	Food	EcM	53	0.3	Boa [1], Eyi-Ndong et al. [18], Degreef et al. [7], De Kesel et al. [4], Milenge et al. [39]	MKH017
14. *Cantharellus floridulus* Heinem.	Food	EcM	54	0.3	De Kesel et al. [16], Boa [1], Eyi-Ndong et al. [18], De Kesel et al. [4], Milenge et al. [39]	ADK5670
15. *Cantharellus incarnatus* (Beeli) Heinem.	Edible	EcM	0	0.0	Boa [1], Eyi-Ndong et al. [18]	MKH069, MKH166
16. *Cantharellus longisporus* Heinem.	Food	EcM	132	0.8	Boa [1], Milenge et al. [39]	ADK5684, MKH141
17. *Cantharellus luteopunctatus* (Beeli) Heinem.	Food	EcM	51	0.3	Boa [1], Eyi-Ndong et al. [18], Milenge et al. [39]	ADK5836, MKH078
18. *Cantharellus miniatescens* Heinem.	Food	EcM	39	0.2	[1], Eyi-Ndong et al. [18], Milenge et al. [39]	ADK5216, MKH142
19. *Cantharellus aff. ruber* Heinem.	Food	EcM	29	0.2	Boa [1], Degreef et al. [7], De Kesel et al. [4], Milenge et al. [39]	ADK5749
20. *Cantharellus rufopunctatus* (Beeli) Heinem.	Food	EcM	84	0.5	Boa [1], Eyi-Ndong et al. [18], De Kesel et al. [4], Milenge et al. [39]	ADK5892, MKH051
21. *Cookeina speciosa* (Fr.) Dennis	Food	Sapr	11	0.1	Eyi-Ndong et al. [18], Milenge et al. [39]	MKH054
22. *Cookeina tricholoma* (Mont.) Kuntze	Edible	Sapr	0	0.0	Boa [1]	ADK5701
23. *Coprinellus disseminatus* (Pers.) J.E. Lange	Food	Sapr	18	0.1	Milenge et al. [39]	ADK5615
24. *Coprinus plicatilis* (Curtis) Fr.	Edible	Sapr	0	0.0	[1]	–
25. *Cotylidia aurantiaca* (Pat.) A. L. Welden	Food	Sapr	14	0.1	Boa [1], Degreef et al. [7], Milenge et al. [39]	ADK5625, MKH058
26. *Dacryopinax spathularia* (Schwein.) G.W. Martin	Edible	Sapr	0	0.0	Boa [1], De Kesel et al. [4]	ADK5578
27. *Favolus tenuiculus* (P. Beauv.) Fr.	Food	Sapr	8	0.1	Eyi-Ndong et al. [18], De Kesel et al. [4], Milenge et al. [39]	MKH109, MKH195
28. *Gerronema hungo* (Henn.) Degreef & Eyi	Edible	Sapr	0	0.0	Eyi-Ndong et al. [18]	MKH057, MKH201
29. *Gymnopilus zenkeri* (Henn.) Singer	Food	Sapr	83	0.5	Eyi-Ndong et al. [18], De Kesel et al. [4], Milenge et al. [39]	ADK5619
30. *Hygrocybe cantharellus* (Schwein.) Murrill	Food	Sapr	28	0.2	Boa [1], Milenge et al. [39]	ADK5849, MKH229
31. *Hygrocybe coccinea* (Schaeff.) P. Kummer	Edible	Sapr	0	0.0	Boa [1]	ADK5864
32. *Hymenagaricus luteolosporus* Heinem.& Little Flower	Food	Sapr	18	0.1	Milenge et al. [39]	ADK5222
33. *Hypholoma subviride* Berk. & M.A. Curtis	Edible	Sapr	0	0.0	Degreef et al. [7]	ADK5891
34. *Lactarius acutus* Heim	Food	EcM	49	0.3	Eyi-Ndong et al. [18], Milenge et al. [39]	ADK5251, MKH001
35. *Lactifluus annulatoangustifolius* (Beeli) Buyck	Food	EcM	28	0.2	Boa [1], Milenge et al. [39]	ADK5862
36. *Lactifluus gymnocarpus* (R. Heim ex Singer) Verbeken	Edible	EcM	0	0.0	Boa [1], Eyi-Ndong et al. [18], De Kesel et al. [4]	MKH135
37. *Lactifluus heimii* (Verbeken) Verbeken	Edible	EcM	0	0.0	Boa [1], De Kesel et al. [4]	MKH170
38. *Lactifluus pelliculatus* (Beeli) Buyck	Edible	EcM	0	0.0	Eyi-Ndong et al. [18], Boa [1]	MKH012
39. *Lentinus squarrosulus* Mont.	Food	Sapr	152	1.0	De Kesel et al. [16], Boa [1], Eyi-Ndong et al. [18], De Kesel et al. [4], Milenge et al. [39]	ADK5226, MKH079
40. *Lepista rhodotoides* Singer	Food	Sapr	54	0.3	Milenge et al. [39]	MKH085
41. *Lepista sordida* (Schumach.) Singer	Edible	Sapr	0	0.0	Boa [1], Degreef et al. [7], De Kesel et al. [4]	ADK5254
42. *Leucocoprinus cepaestipes* (Sowerby) Pat	Food	Sapr	19	0.1	Boa [1], Milenge et al. [39]	ADK5206
43. *Leucocoprinus discoideus* (Beeli) Heinem	Edible	Sapr	0	0.0	Boa [1]	ADK5606
44. *Macrolepiota dolichaula* (Berk. & Broome) Pegler & R.W. Rayner	Edible	Sapr	0	0.0	Boa [1], Eyi-Ndong et al. [18], Degreef et al. [7], De Kesel et al. [4]	ADK5207, MKH223
45. *Marasmiellus inoderma* (Berk.) Singer	Edible	Sapr	0	0.0	Eyi-Ndong et al. [18], Degreef et al. [7]	ADK5737, MKH094
46. *Marasmius arborescens* (Henn.) Beeli	Food	Sapr	99	0.6	Boa [1], Eyi-Ndong et al. [18], [7], De Kesel et al. [4], Milenge et al. [39]	ADK5255, MKH231
47. *Marasmius bekolacongoli* Beeli	Food	Sapr	62	0.4	Eyi-Ndong et al. [18], Degreef et al. [7], De Kesel et al. [4], Milenge et al. [39]	ADK5779, MKH035
48. *Marasmius buzungolo* Singer	Food	Sapr	138	0.9	Boa [1], Eyi-Ndong et al. [18], Milenge et al. [39]	ADK5760, MKH121
49. *Marasmius confertus* Berk. & Broome	Food	Sapr	51	0.3	Milenge et al. [39]	ADK5276, MKH021
50. *Neonothopanus hygrophanus* (Mont.) De Kesel & Degreef	Edible	Sapr	0	0.0	Eyi-Ndong et al. [18], De Kesel et al. [4]	ADK5260, MKH031
51. *Paxillus brunneotomentosus* Heinem. & Rammeloo	Edible	Sapr	0	0.0	Degreef et al. [7]	ADK5655, MKH129
52. *Pleurotus cystidiosus* O.K. Mill.	Food	Sapr	118	0.7	De Kesel et al. [16], Boa [1], Degreef et al. [7], Milenge et al. [39]	MKH067
53. *Pleurotus flabellatus* (Berk. & Br.) Sacc.	Food	Sapr	48	0.3	Eyi-Ndong et al. [18], Degreef et al. [7], Milenge et al. [39]	ADK5771
54. *Pleurotus tuber-regium* (Fr.) Fr.	Food	Sapr	98	0.6	De Kesel et al. [16], Boa [1], Eyi-Ndong et al. [18], Degreef et al. [7], De Kesel et al. [4], Milenge et al. [39]	MKH106
55. *Polyporus arcularius* (Batsch) Fr.	Edible	Sapr	0	0.0	Boa [1]	MKH107
56. *Psathyrella candolleana* (Fr.) Maire	Edible	Sapr	0	0.0	Boa [1]	ADK5252
57. *Rubinoboletus luteopurpureus* (Beeli) Heinem. & Rammeloo	Food	EcM	24	0.2	Boa [1], Milenge et al. [39]	ADK5192, MKH235
58. *Russula annulata* R. Heim	Food	EcM	32	0.2	Milenge et al. [39]	MKH014
59. *Russula inflata* Buyck	Food	EcM	29	0.2	Milenge et al. [39]	ADK5217
60. *Russula meleagris* Buyck	Food	EcM	35	0.2	Boa [1], Milenge et al. [39]	MKH113
61. *Russula porphyrocephala* Buyck	Food	EcM	26	0.2	Milenge et al. [39]	ADK5750, MKH178
62. *Russula pruinata* Buyck	Food	EcM	27	0.2	Milenge et al. [39]	MKH115, MKH151
63. *Russula roseostriata* Buyck	Edible	EcM	0	0.0	Boa [1], Eyi-Ndong et al. [18]	MKH150
64. *Russula sese* Beeli	Edible	EcM	0	0.0	Boa [1], Eyi-Ndong et al. [18]	MKH114
65. *Russula sesemoindu* Beeli	Food	EcM	49	0.3	Eyi-Ndong et al. [18], Milenge et al. [39]	MKH146
66. *Russula striatoviridis* Buyck	Food	EcM	52	0.3	Boa [1], Eyi-Ndong et al. [18], Milenge et al. [39]	ADK5896
67. *Schizophyllum commune* Fr.	Food	Sapr	160	1.0	De Kesel et al. [16], Boa [1], Eyi-Ndong et al. [18], Degreef et al. [7], De Kesel et al. [4], Milenge et al. [39]	MKH108
68. *Tapinella panuoides* (Fr.) E.-J. Gilbert	Food	Sapr	26	0.2	Milenge et al. [39]	MKH043
69. *Termitomyces robustus* (Beeli) Heim	Food	Term	128	0.8	De Kesel et al. [16], Boa [1], Eyi-Ndong et al. [18], Degreef et al. [7], De Kesel et al. [4], Milenge et al. [39]	ADK5242
70. *Termitomyces singidensis* Saarim. & Härk.	Food	Term	66	0.4	Boa [1]	ADK5852
71. *Termitomyces striatus* (Beeli) Heim	Food	Term	124	0.8	De Kesel et al. [16], Boa [1], Eyi-Ndong et al. [18], Degreef et al. [7], De Kesel et al. [4]	ADK5179
72. *Tremella fuciformis* Berkeley	Edible	Sapr	0	0.0	Boa [1]	MKH047
73. *Tricholomopsis aurea* (Beeli) Desjardin & B.A. Perry.	Food	Sapr	64	0.4	Boa [1], Eyi-Ndong et al. [18]	ADK5625, MKH154
74. *Trogia infundibuliformis* Berk. & Br.	Food	Sapr	38	0.2	Eyi-Ndong et al. [18], De Kesel et al. [4], Milenge et al. [39]	ADK5223
75. *Tylopilus balloui* (Peck) Singer	Edible	EcM	0	0.0	Boa [1]	ADK5950
76. *Volvariella parvispora* Heinem.	Edible	Sapr	0	0.0	Boa [1], De Kesel et al. [4]	ADK5786
77. *Volvopluteus gloiocephalus* (DC) Vizzini	Edible	Sapr	0	0.0	Boa [1]	ADK5641
78. *Xerocomus spinulosus* Heinem. & Gooss.-Font.	Food	EcM	12	0.1	De Kesel et al. [4], Milenge et al. [39]	ADK5209

Edible = species known as edible but not reported locally consumed, food = species locally consumed, *FM* frequency of mention

### Trophic groups and distribution of edible fungi

**Fig. 2:**
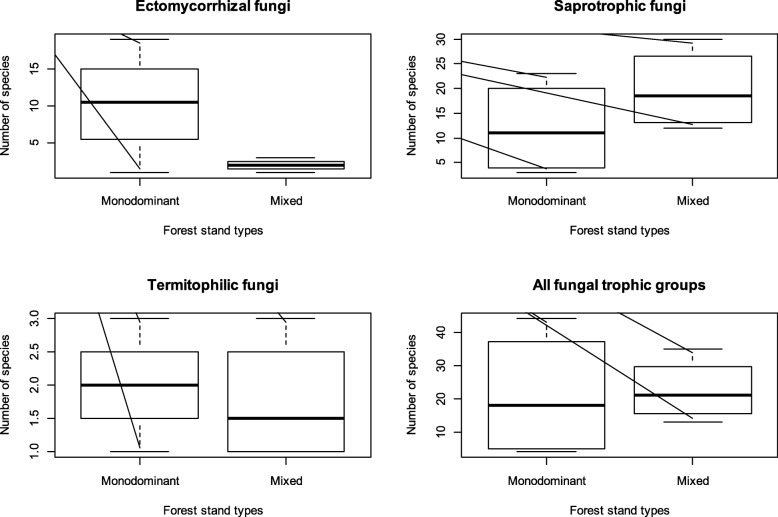
Diversity of edible fungi according to trophic groups and forest stands

**Fig. 3:**
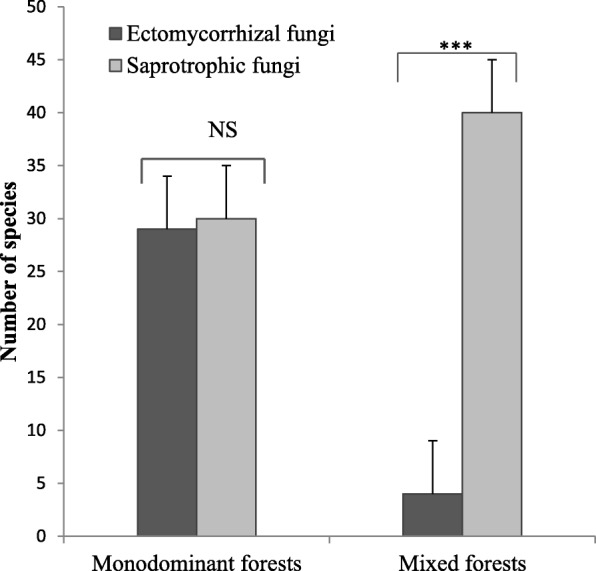
Number of ectomycorrhizal and saprotrophic fungi within monodominant and mixed forests. NS no significant difference; ***highly significant difference

Edible species belong to 3 trophic groups (saprotrophic, ectomycorrhizal, and termite associated). The saprotrophic taxa (wood and litter decaying fungi) represent the most species-rich group (44 species), followed by the EcM fungi (31 species) and termite-associated taxa (3 species, all *Termitomyces*). Whereas the number of edible fungi significantly differs between trophic groups, strong variation in the composition of edible fungi is observed between forest types (Fig. 2). Figure 2 shows that the mixed forests, on average, hold the highest diversity in saprotrophic edible fungi, while EcM fungi reach their highest species numbers in the monodominant forests. Edible termite-associated fungi (*Termitomyces* sp.) show the lowest species numbers and seem equally represented in both mixed and monodominant forests. Considering all fungal trophic groups, plots from monodominant and mixed forests showed similar average numbers of edible species, but variations are more important in the monodominant forests. The result is that the highest numbers of edible species were encountered in some of the monodominant forests, especially in the forest dominated by *Gilbertiodendron dewevrei*. Within monodominant forests, we observed no significant difference in the number of saprotrophic and EcM fungi (Fig. 3). In contrast, within mixed forests, the number of saprotrophic taxa was significantly higher than in the EcM forests.

### Usage of edible fungi

Almost a third (25 species) of the 78 edible species are not used for food by any of the interviewed people (Table 2). This is the case for *Cantharellus conspicuus*, *C. incarnatus*, *Cookeina tricholoma*, *Coprinus plicatilis*, *Dacryopinax spathularia*, *Gerronema hungo*, *Hygrocybe coccinea*, *Hypholoma subviride*, *Lactifluus gymnocarpus*, *L. heimii*, *L. pelliculatus*, *Lepista sordida*, *Leucocoprinus discoideus*, *Macrolepiota dolichaula*, *Marasmiellus inoderma*, *Neonothopanus hygrophanus*, *Paxillus brunneotomentosus*, *Polyporus arcularius*, *Psathyrella candolleana*, *Russula roseostriata*, *R. sese*, *Tremella fuciformis*, *Tylopilus balloui*, *Volvariella parvispora*, and *Volvopluteus gloiocephalus*.

**Table 2:** Edible fungi local names and list of fungi eaten by informants from each ethnic group

Species	Local names	Ethnic groups					
		Kumu	Lokele	Ngando	Ngelema	Topoke	Turumbu
*Agaricus bambusicola* Heinem.	NA	−	+	+	+	+	+
*Agaricus crocopeplus* Berk. & Broome	NA	−	+	+	+	+	+
*Amanita annulatovaginata* Beeli	NA	−	+	+	+	+	+
*Amanita echinulata* Beeli	NA	+	+	+	+	+	+
*Amanita pudica* (Beeli) Walleyn	NA	+	+	+	+	+	+
*Amanita robusta* Beeli	Balenda^1^	+	+	+	+	+	+
*Armillaria heimii* Pegler	Baselekoko^3^	+	+	+	+	+	+
*Auricularia cornea* Ehrenb.	Batele^1,2,3,4,5,6^	+	+	+	+	+	+
*Auricularia delicata* (Mont.ex Fr.) Henn.	Batele^1,2,3,4,5,6^	+	+	+	+	+	+
*Calyptella longipes* (Cooke & Massee) W.B. Cooke	Bape-bape^6^	−	+	+	−	+	+
*Cantharellus congolensis* Beeli	Bolingobopii^6^	+	+	+	+	+	+
*Cantharellus conspicuus* Eyssartier, Buyck & Verbeken	NA	−	−	−	−	−	−
*Cantharellus densifolius* Heinem.	NA	+	+	+	+	+	+
*Cantharellus floridulus* Heinem.	Solosolo^3^	+	+	+	+	+	+
*Cantharellus incarnatus* (Beeli) Heinem.	NA	−	−	−	−	−	−
*Cantharellus longisporus* Heinem.	Baolo-bandei^6^, Abamongo^1^	+	+	+	+	+	+
*Cantharellus luteopunctatus* (Beeli) Heinem.	Baolo-bandei^6^, Abamongo^1^	+	+	+	+	+	+
*Cantharellus miniatescens* Heinem.	Lilenda-linenu^6^, Agbalo-gbalo^4^	+	+	+	+	+	+
*Cantharellus aff. ruber* Heinem.	NA	+	+	+	+	+	+
*Cantharellus rufopunctatus* (Beeli) Heinem.	Bengole-bonyele^6^	+	+	+	+	+	+
*Cookeina speciosa* (Fr.) Dennis	Batoyi bangwa’a^2^	−	−	+	−	−	+
*Cookeina tricholoma* (Mont.) Kuntze	Batoyi bangwa’a^2^	−	−	−	−	−	−
*Coprinellus disseminatus* (Pers.) J.E. Lange	Baku’kulekule^6^	−	+	+	+	+	+
*Coprinus plicatilis* (Curtis) Fr.	NA	−	−	−	−	−	−
*Cotylidia aurantiaca* (Pat.) A. L. Welden	NA	−	−	+	−	−	+
*Dacryopinax spathularia* (Schwein.) G.W. Martin	NA	−	−	−	−	−	−
*Favolus tenuiculus* (P. Beauv.) Fr.	NA	−	−	+	−	−	+
*Gerronema hungo* (Henn.) Degreef & Eyi	NA	−	−	−	−	−	−
*Gymnopilus zenkeri* (Henn.) Singer	Bokoo’kina^6^	+	+	+	+	+	+
*Hygrocybe cantharellus* (Schwein.) Murrill	Adanopila^4^	−	+	+	+	+	+
*Hygrocybe coccinea* (Schaeff.) P. Kummer	NA	−	−	−	−	−	−
*Hymenagaricus luteolosporus* Heinem*.*& Little Flower	Baolo ba’ongolo^6^, Tongwengwe^4^	−	+	+	+	+	+
*Hypholoma subviride* Berk. & M.A. Curtis	NA	−	−	−	−	−	−
*Lactarius acutus* Heim	Mongo^5^	−	+	+	+	+	+
*Lactifluus annulatoangustifolius* (Beeli) Buyck	Belaa’tala^3^	−	+	+	+	+	+
*Lactifluus gymnocarpus* (R. Heim ex Singer) Verbeken	NA	−	−	−	−	−	−
*Lactifluus heimii* (Verbeken) Verbeken	NA	−	−	−	−	−	−
*Lactifluus pelliculatus* (Beeli) Buyck	Batina lya bakuu^6^	−	−	−	−	−	−
*Lentinus squarrosulus* Mont.	Bengole^6^	+	+	+	+	+	+
*Lepista rhodotoides* Singer	Bongolototo^6^, Baselesele^2^	−	+	+	+	+	+
*Lepista sordida* (Schumach.) Singer	NA	−	−	−	−	−	−
*Leucocoprinus cepaestipes* (Sowerby) Pat	Mbumbuke^6^	−	+	+	−	+	+
*Leucocoprinus discoideus* (Beeli) Heinem	NA	−	−	−	−	−	−
*Macrolepiota dolichaula* (Berk. & Broome) Pegler & R.W. Rayner	NA	−	−	−	−	−	−
*Marasmiellus inoderma* (Berk.) Singer	NA	−	−	−	−	−	−
*Marasmius arborescens* (Henn.) Beeli	Twendenda^5^, Seeliko^3^	+	+	+	+	+	+
*Marasmius bekolacongoli* Beeli	Bakoko wa kombe^6^, Toko kombe^2^	+	+	+	+	+	+
*Marasmius buzungolo* Singer	Mongala^6^, Ndundo^3,5^	+	+	+	+	+	+
*Marasmius confertus* Berk. & Broome	Mongala^6^	+	+	+	+	+	+
*Neonothopanus hygrophanus* (Mont.) De Kesel & Degreef	NA	−	−	−	−	−	−
*Paxillus brunneotomentosus* Heinem. & Rammeloo	NA	−	−	−	−	−	−
*Pleurotus cystidiosus* O.K. Mill.	Bengole^6,2,5^	+	+	+	+	+	+
*Pleurotus flabellatus* (Berk. & Br.) Sacc.	Bengole^6,2,5^	+	+	+	+	+	+
*Pleurotus tuber-regium* (Fr.) Fr.	Bengole ya makasi^6^	+	+	+	+	+	+
*Polyporus arcularius* (Batsch) Fr.	NA	−	−	−	−	−	−
*Psathyrella candolleana* (Fr.) Maire	NA	−	−	−	−	−	−
*Rubinoboletus luteopurpureus* (Beeli) Heinem. & Rammeloo	Lofyongi lonyele^6^	−	+	+	+	+	+
*Russula annulata* R. Heim	Lilianga^6^	−	+	+	+	+	+
*Russula inflata* Buyck	Balenda ya moindo^6^	−	+	+	−	+	+
*Russula meleagris* Buyck	Nsalanka’ngaa^6^	+	+	+	+	+	+
*Russula porphyrocephala* Buyck	Nsololo^6^	+	+	+	+	+	+
*Russula pruinata* Buyck	Balenda ya kulokoko^6^	−	+	+	+	+	+
*Russula roseostriata* Buyck	NA	−	−	−	−	−	−
*Russula sese* Beeli	NA	−	−	−	−	−	−
*Russula sesemoindu* Beeli	Malebadja^6^	−	+	+	+	+	+
*Russula striatoviridis* Buyck	NA	−	+	+	−	+	+
*Schizophyllum commune* Fr.	Bokotoko^6^, Tukumu^2^, Bokokola^1,3,4,5^	+	+	+	+	+	+
*Tapinella panuoides* (Fr.) E.-J. Gilbert	Tukutuno^6^	−	+	+	−	+	+
*Termitomyces robustus* (Beeli) Heim	Limusula^6^, Libusula^5^, Bululu^3^, Nku’suusu^1^	+	+	+	+	+	+
*Termitomyces singidensis* Saarim. & Härk.	Limusula^6^, Libusula^5^, Bululu^3^	−	+	+	−	+	+
*Termitomyces striatus* (Beeli) Heim	Limusula^6^, Libusula^5^, Bululu^3^	+	+	+	+	+	+
*Tremella fuciformis* Berkeley	NA	−	−	−	−	−	−
*Tricholomopsis aurea* (Beeli) Desjardin & B.A. Perry.	NA	+	+	+	+	+	+
*Trogia infundibuliformis* Berk. & Br.	NA	+	+	+	+	+	+
*Tylopilus balloui* (Peck) Singer	NA	−	−	−	−	−	−
*Volvariella parvispora* Heinem.	NA	−	−	−	−	−	−
*Volvopluteus gloiocephalus* (DC) Vizzini	NA	−	−	−	−	−	−
*Xerocomus spinulosus* Heinem. & Gooss.-Font.	Kwekwele^6^	−	−	+	−	−	+

^1^Kumu, ^2^Lokele, ^3^Ngando, ^4^Ngelema, ^5^Topoke, ^6^Turumbu (mother tongues); “+” indicates edible fungi and “−” indicates inedible fungi by local ethnic groups’ informants; *NA* not applicable

**Fig. 4:**
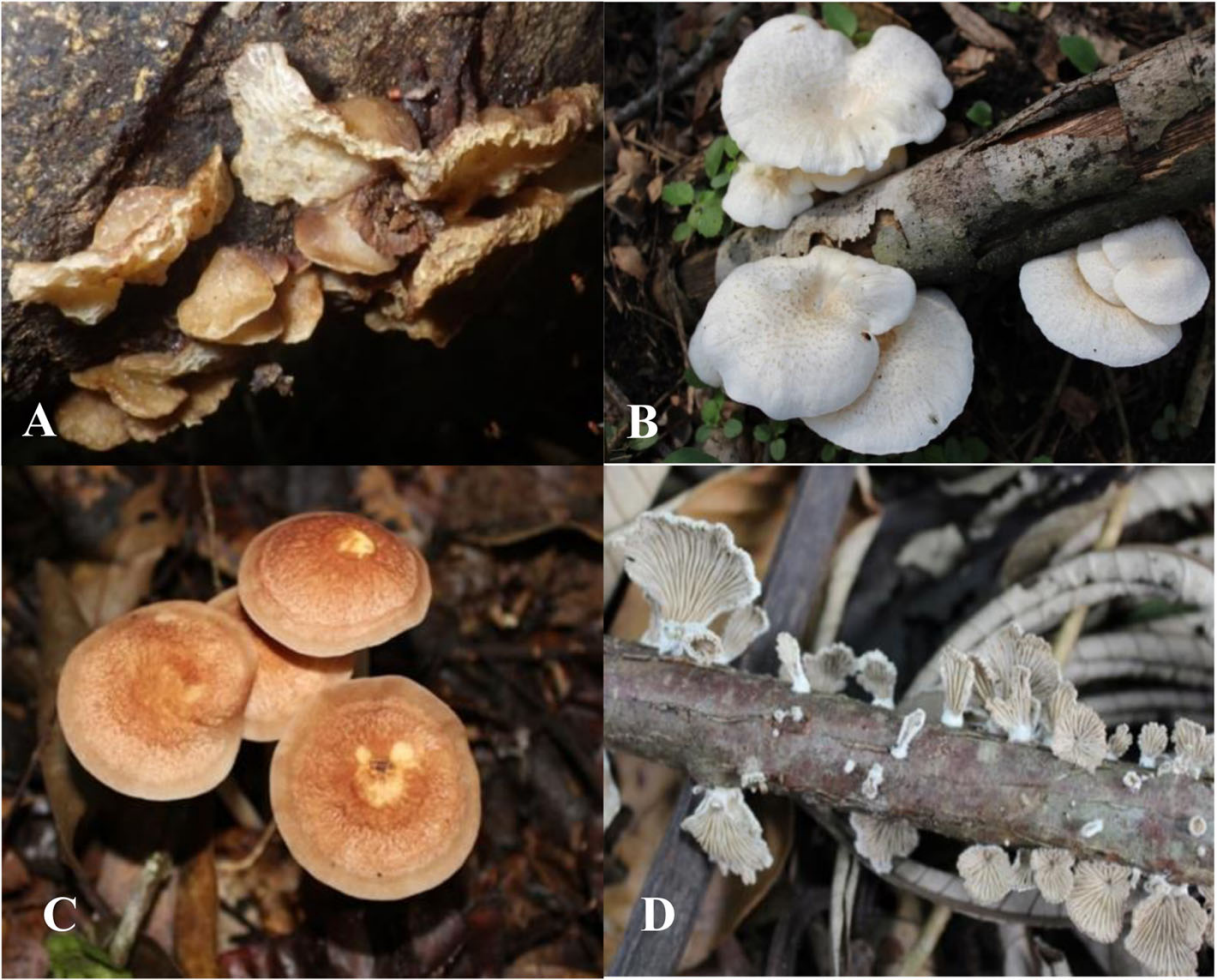
Four of the most important edible saprotrophic fungi in Tshopo province. **a***Auricularia delicata*. **b***Lentinus squarrosulus*. **c***Marasmius buzungolo*. **d***Schizophyllum commune*

Significant differences were observed in the mean number of ectomycorrhizal and saprotrophic fungi eaten by informants from different ethnic groups (Table 3). Because of their low numbers (three species), differences in the mean numbers of consumed termite-associated species are evidently small. In general, the most commonly eaten mushrooms (with the highest edibility index) are saprotrophic or woody-decaying fungi (Table 3), the most appreciated ones being *Auricularia* spp*.*, *Marasmius buzungolo*, *Lentinus squarrosulus*, and *Schizophyllum commune* (Fig. 4). Only a small fraction of EcM fungi, especially from the genus *Cantharellus*, are reported as a delicacy by some populations. The scores of EcM fungi are even more lowered, knowing that some local populations (Kumu) systematically reject all edible *Russula* because of their vivid colors. In terms of diversity and quantity, local markets (Yangambi) offer far more saprotrophic than EcM fungi (De Kesel pers. obs.).

**Table 3:** Mean numbers of edible fungi eaten by informants from each ethnic group (tribe) ± standard deviation (SD)

Ethnic groups	Fungal trophic groups			Kruskal-Wallis	
	Ectomycorrhizal fungi, mean ± SD	Saprotrophic fungi, mean ± SD	Termitophilic fungi, mean ± SD		
				*p* value	Significance
Turumbu	8 ± 3	13 ± 3	2 ± 1	< 0.001	***
Lokele	7 ± 3	12 ± 3	2 ± 1	< 0.001	***
Topoke	7 ± 3	13 ± 3	2 ± 1	< 0.001	***
Ngando	19 ± 3	24 ± 4	2 ± 1	< 0.001	***
Ngelema	10 ± 3	12 ± 2	2 ± 1	< 0.001	***
Kumu	8 ± 2	11 ± 2	2 ± 1	< 0.001	***
*p* value	< 0.001	< 0.001	> 0.05		
Significance	***	***	NS		

The two last columns and lines indicate the *p* value and significance level of the Kruskal-Wallis test, respectively (*NS* no significant difference, ***very high significant difference)

## Discussion

This study revealed that wild edible fungi from rainforests of Tshopo belong to several trophic groups. They can be either saprotrophic (growing on dead fallen trunks and litter), or termite associated (growing in mutualistic relation with termites), or ectomycorrhizal, i.e., living in a mutualistic relation with the roots of some vascular plants. Within rainforests from the Yangambi Biosphere reserve and Yoko reserve, relatively high numbers of edible fungi are available. A more quantitative approach, similar to the one used by Yorou et al. [41] or De Kesel et al. [4], is needed to ascertain this, but it seems that the EcM fraction of edible fungi is still underexploited. Although a wide array of edible EcM fungi is available in the primary ectomycorrhizal forests, the interest of local people mainly goes to saprotrophic fungi from the degraded mixed forests. Milenge et al. [39] reported that most of these saprotrophic edible taxa typically grow on wood (logs).

Among the reported edible EcM fungi, only species of the genus *Cantharellus* are considered of some importance in local consumption [39]. As stated by Eyi-Ndong et al. [18], species of the genus *Cantharellus* are the most important edible EcM fungi found within rainforests from the central African Congo basin. Most species of *Cantharellus* are known worldwide as good or even excellent edibles. With its 42 African endemic species, the genus is by far best represented on the African continent [9]. In contrast to the mushroom diet chosen by people from Tshopo, *Cantharellus* species are massively consumed in the Zambezian region [4, 5, 9, 10, 13, 14, 42].

Although other EcM genera, i.e., *Amanita*, *Lactarius*, *Lactifluus*, and *Russula*, are common in the forests dominated by *Gilbertiodendron dewevrei*, these edible EcM fungi do not seem to be at all harvested and consumed by local populations of the Tshopo province. However, in miombo areas of Burundi [7, 8, 43], and similar woodlands in the Zambezian [4, 11] and Guineo-Soudanian ecozone [15–17], these genera (and *Cantharellus*) represent a major source of food and income.

Triggering the interest of local people for non-timber forest products requires science-based information about how much of the resource is available, how much and where it can be obtained in a sustainable way, and what its value may be. Based on productivity data of the 50 most common edible species, 1 ha of Katangese miombo woodland produces an average of 148 kg of fresh fruiting bodies per year [4]. A very similar biomass, i.e., 140.7 kg/ha/year, was obtained from 29 edible species monitored in the savanna woodlands of Benin [17]. From miombo woodlands of Katanga in the Democratic Republic of the Congo for instance, 1 kg of fresh fruiting bodies of *Cantharellus* spp. costs approximatively 2.8 USD [4]. Local people realize that the yearly revenue obtained from these NTFP goes hand in hand with sustainable use and preservation of host trees and their immediate environment. Losing revenue from not being able to collect EcM fungi is a strong incentive to preserve, or at least make sure, that the forest ecosystem keeps on delivering its services.

In this context, it surely is interesting to monitor natural productions of edible EcM fungi in different types of rainforests of the Democratic Republic of the Congo. So far, this has not been done. Gathering such data is one thing, but convincing local people of changing or widening their diet, i.e., to include more EcM fungi, is another. Several scientists [7, 17, 27, 44–47] have reported the role of culture and tradition on the variety of mushrooms consumed by local people. In the context of the province of Tshopo, taboos resting on mushroom consumption create mycophobia and subsequently decrease the interest for some edible EcM species. In spite of their high nutritional value, most *Russula* species are not eaten by Kumu people because of vivid colors of the cap. Variation in mushroom consumption may also rely on the scarcity of the fruiting bodies [7], and general appreciation is bound to be affected by availability and ease of access to the resource. When some taxa are rare or only available in more remote areas, people may find it difficult to go far to collect them. The result is that mushroom diets can and will shift as a mere result of access and distance to suitable sites [7].

We have no data from eventual hunter-gatherers in the region, but the main reason why people from the Tshopo show a preference for saprotrophic fungi is that these species are found close to the villages and nearby recently cut forests. Because of frequent rains and availability of dead wood, saprotrophic taxa also manage to produce fruit bodies all year round. In contrast, EcM fungi occur abundantly in remote and pristine (or nearly pristine) forests with older growth of *Gilbertiodendron dewevrei*, *Brachystegia laurentii*, or *Uapaca* spp. [19]. With the exception of *Cantharellus rufopunctatus*, producing fruit bodies at least from April to December [9], most of the edible EcM taxa can only be found during a short period of time.

## Conclusion

The results from this study reveal that rainforests from the Yangambi Biosphere reserve and Yoko forest reserve of the Tshopo provide a substantial number of edible fungi. Although not formally measured, there is little doubt that the local populations collect and use only a small fraction of the naturally produced quantity of edible fungi. Most of the locally used taxa are saprotrophic and easily accessed from either litter or dead wood. It is the availability, abundance, and proximity of this resource that makes saprotrophic fungi from anthropized environments (secondary forests, clearings, fallow, farmland) most attractive and preferred. As EcM fungi are harder to obtain and less frequent, they are a poorly known and underutilized food source. Whereas in the Zambezian region, the major local interest for edible EcM fungi can be used as a strong incentive for forest conservation; our observations indicate that this is not the case in the Tshopo region.

Some locally considered inedible EcM fungal taxa are in fact perfectly edible and used for food elsewhere in Africa. More research needs to be done, but assessing the natural productivity of edible fungi from the rainforests should help us better understand what these fungi can mean as an ecosystem service to local livelihoods. In combination with the measured interest of local people for this resource, one should be able to develop systems to promote the use of this largely untapped resource.

## Data Availability

The data that support the findings of this study have been deposited at the herbarium of the Meise Botanic Garden in Belgium (BR) through the reference codes ADK (for the collection made by André De Kesel) and MKH (for the collection made by Milenge Kamalebo Héritier). Some data are accessible on https://www.efta-online.org/. The overall data are available from the authors.

## Abbreviations

DR Congo: Democratic Republic of the Congo
EcM: Ectomycorrhizal (fungi, forests, trees)
EMCS: Edible mushrooms’ cultural significance
NTFP: Non-timber forest product
Sapr: Saprotrophic fungi
Term: Termitophilic fungi
